# Carbapenem- and cefiderocol-resistant *Enterobacterales* in surface water in Kumasi, Ashanti Region, Ghana

**DOI:** 10.1093/jacamr/dlae021

**Published:** 2024-03-06

**Authors:** Elias Eger, Timo Homeier-Bachmann, Eugene Adade, Sylvia Dreyer, Stefan E Heiden, Phillip Lübcke, Patrick O Tawiah, Augustina A Sylverken, Sascha Knauf, Katharina Schaufler

**Affiliations:** Department of Epidemiology and Ecology of Antimicrobial Resistance, Helmholtz Institute for One Health, Helmholtz Centre for Infection Research HZI, Greifswald, Germany; Institute of Epidemiology, Friedrich-Loeffler-Institut, Federal Research Institute for Animal Health, Greifswald—Insel Riems, Germany; Department of Theoretical and Applied Biology, Kwame Nkrumah University of Science and Technology, Kumasi, Ghana; Kumasi Centre for Collaborative Research in Tropical Medicine, Kwame Nkrumah University of Science and Technology, Kumasi, Ghana; Institute of International Animal Health/One Health, Friedrich-Loeffler-Institut, Federal Research Institute for Animal Health, Greifswald—Insel Riems, Germany; Department of Epidemiology and Ecology of Antimicrobial Resistance, Helmholtz Institute for One Health, Helmholtz Centre for Infection Research HZI, Greifswald, Germany; Pharmaceutical Microbiology, Institute of Pharmacy, University of Greifswald, Greifswald, Germany; Department of Theoretical and Applied Biology, Kwame Nkrumah University of Science and Technology, Kumasi, Ghana; Kumasi Centre for Collaborative Research in Tropical Medicine, Kwame Nkrumah University of Science and Technology, Kumasi, Ghana; Department of Theoretical and Applied Biology, Kwame Nkrumah University of Science and Technology, Kumasi, Ghana; Kumasi Centre for Collaborative Research in Tropical Medicine, Kwame Nkrumah University of Science and Technology, Kumasi, Ghana; Institute of International Animal Health/One Health, Friedrich-Loeffler-Institut, Federal Research Institute for Animal Health, Greifswald—Insel Riems, Germany; Faculty of Veterinary Medicine, Justus-Liebig-University, Giessen, Germany; Department of Epidemiology and Ecology of Antimicrobial Resistance, Helmholtz Institute for One Health, Helmholtz Centre for Infection Research HZI, Greifswald, Germany; Pharmaceutical Microbiology, Institute of Pharmacy, University of Greifswald, Greifswald, Germany; University Medicine Greifswald, Greifswald, Germany

## Abstract

**Background:**

MDR pathogens including ESBL- and/or carbapenemase-producing *Enterobacterales* (ESBL-PE and CPE) increasingly occur worldwide in the One Health context.

**Objectives:**

This proof-of-principle study investigated the occurrence of ESBL-PE in surface water in the Ashanti Region in Ghana, sub-Saharan Africa (SSA), and investigated their additional genotypic and phenotypic antimicrobial resistance features as part of the Surveillance Outbreak Response Management and Analysis System (SORMAS).

**Methods:**

From 75 water samples overall, from nine small to medium-sized river streams and one pond spatially connected to a channelled water stream in the greater area of Kumasi (capital of the Ashanti Region in Ghana) in 2021, we isolated 121 putative ESBL-PE that were subsequently subjected to in-depth genotypic and phenotypic analysis.

**Results:**

Of all 121 isolates, *Escherichia coli* (70.25%) and *Klebsiella pneumoniae* (23.14%) were the most prevalent bacterial species. In addition to ESBL enzyme-production of mostly the CTX-M-15 type, one-fifth of the isolates carried carbapenemase genes including *bla*_NDM-5_. More importantly, susceptibility testing not only confirmed phenotypic carbapenem resistance, but also revealed two isolates resistant to the just recently approved last-resort antibiotic cefiderocol. In addition, we detected several genes associated with heavy metal resistance.

**Conclusions:**

ESBL-PE and CPE occur in surface water sources in and around Kumasi in Ghana. Further surveillance and research are needed to not only improve our understanding of their exact prevalence and the reservoir function of water sources in SSA but should include the investigation of cefiderocol-resistant isolates.

## Introduction

Antimicrobial resistance (AMR) has emerged as an urgent global health crisis, severely limiting available therapeutic options including last-resort antibiotics such as carbapenems. Sub-Saharan Africa (SSA) bears a high burden of AMR, with a significant number of deaths attributed to ESBL- and carbapenemase-producing *Enterobacterales* (ESBL-PE and CPE) such as *Escherichia coli* and *Klebsiella pneumoniae*.^1^ In Ghana, multiple studies have revealed an increasing number of hospital- and community-associated infections with ESBL producers.^2^ Like many other low- and middle-income countries (LMICs), Ghana faces challenges regarding unclean water due to open drains, limited sanitation and healthcare, and a lack of public education and environmental consciousness. While there is evidence suggesting that environmental sources might serve as potential transmission points for AMR bacteria in SSA, the extent of environmental contamination including surface water with ESBL-PE and CPE remains largely unknown. One of the few available studies revealed overall high rates of ESBL-producing *E. coli* in rivers in two cities in Ghana between 2018 and 2020.^3^ In a broader African context, data on the presence of ESBL-PE and particularly CPE in environmental settings across the continent are exceptionally scarce. In addition, nothing is known about the occurrence of *Enterobacterales* resistant to the recently approved antibiotic cefiderocol in these niches.

This proof-of-principle study’s objective was to complement the Surveillance Outbreak Response Management and Analysis System (SORMAS) project^4^ with data on the occurrence of ESBL-PE and CPE in surface water from Kumasi, the capital city of the Ashanti Region in Ghana.

## Methods

### Sampling strategy and bacterial isolation

**Figure 1.**
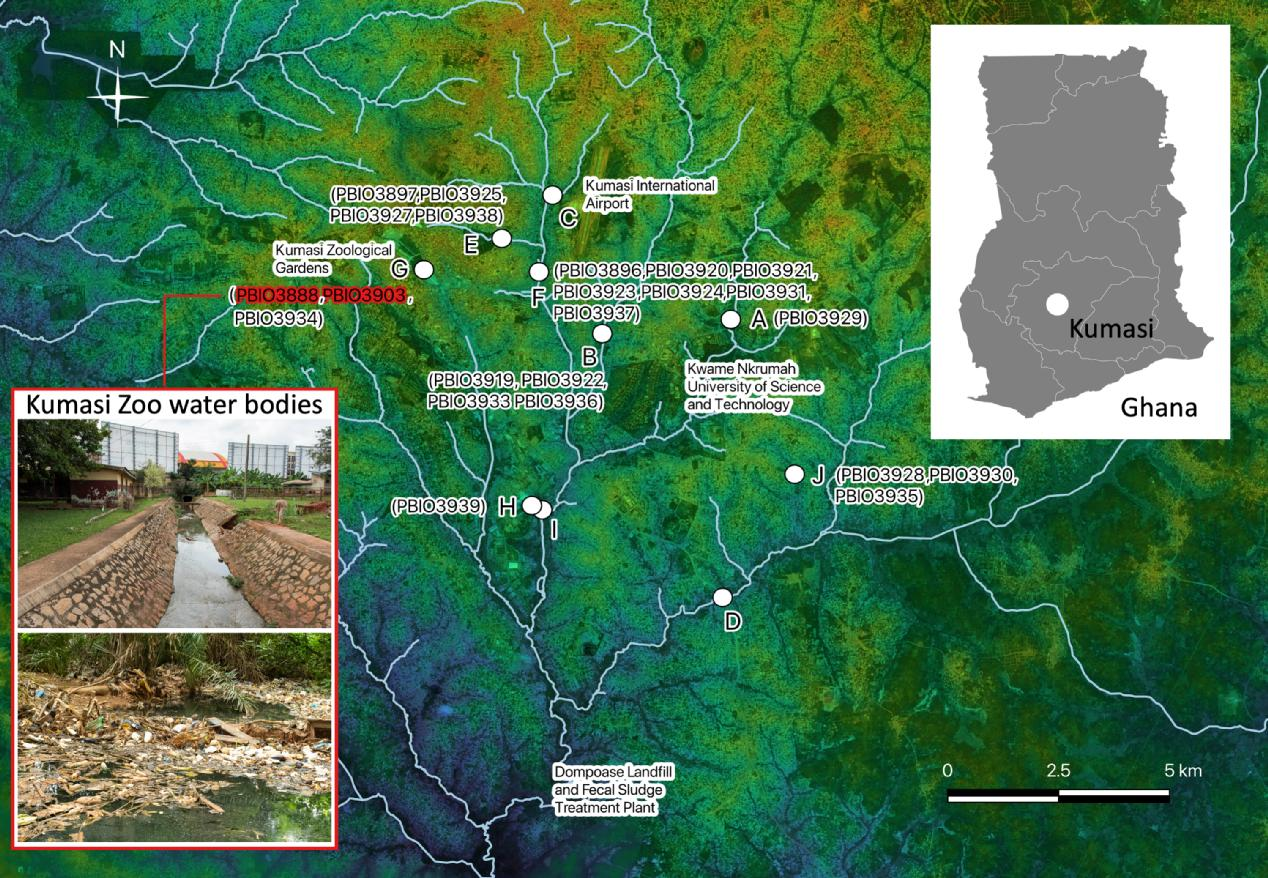
Map of the Kumasi area (Ghana). The selected water sources are labelled from (A) to (J). Sample sizes were (A) *n* = 8, (B) *n* = 8, (C) *n* = 8, (D) *n* = 8, (E) *n* = 7, (F) *n* = 7, (G) *n* = 7, (H) *n* = 7, (I) *n* = 7 and (J) *n* = 8. The CPE isolates are assigned to their sampling locations. The inset image outlined in red shows images of the Kumasi Zoo water bodies (location G) where the two cefiderocol-resistant isolates (highlighted in red) were isolated. The map was created in QGIS v. 3.20 using Bing Aerial (http://ecn.t3.tiles.virtualearth.net/tiles/a{q}.jpeg?g=1) as a map source with TM World Borders 0.3 overlay (https://thematicmapping.org/downloads/world_borders.php; last accessed on 23 January 2024). Microsoft product screenshots were reprinted with permission from Microsoft Corporation. Major surface waters are highlighted in pale blue based on the OpenStreetMap data for Ghana (CC BY-SA 2.0 DEED; https://download.geofabrik.de/africa/ghana.html). The Digital Elevation Model (DEM) layer uses a single band with values from 185.2 to 388.5. For reference, the overall location of Kumasi is indicated on the Ghana overview map with Regions (https://vemaps.com/ghana/gh-04 by Vemaps.com).

We collected 75 surface water samples from 10 randomly chosen peri-urban sites in the Kumasi area in Ghana (Figure 1). This included nine small- to medium-sized river streams and one pond spatially connected to a channelled water stream. Note that the individual sampling spots are representative of the variety of water sources found in the Kumasi area, which are used by animals and humans alike. Observed activities around the water bodies included irrigation and collection of water for households, laundry and livestock. Sampling of 1 L water volume per site was performed once a week over 7–8 weeks between July and September 2021. Water samples were collected directly by filling a sterile plastic container with surface water. Samples were refrigerated at +4°C and further processed at the laboratory of the Kumasi Centre for Collaborative Research, Ghana, within 4 h of collection. Samples were then prefiltered using a sterile gauze (PZN: 04046708, FESMED Verbandmittel GmbH, Frankenberg/Sa., Germany) to retain macroscopic particles. This was followed by filtration through a sterile 1.20 µm, and ultimately 0.45 µm, pore-sized membrane filter (Millipore, Merck, Darmstadt, Germany). Filtration was supported by the ‘All-Glass Filter Holder Kit’ (XX5514700) combined with a pump (WP6122050, 220 V/50 Hz, both from Millipore). Similar to a previous study,^5^ a 6 mm-sized piece of the 0.45 µm filter was transferred to 10 LB broths (Lennox, Sigma–Aldrich, Merck KGaA, Darmstadt, Germany) containing 2 µg/mL cefotaxime (VWR International, Darmstadt, Germany) and cultured at 37°C and 200 rpm overnight. On the following day, 1.8 mL of the culture was pelleted at 20 000×**g** for 1 min and resuspended in 1 mL of the same culture medium supplemented with glycerol (anhydrous; Merck, Darmstadt, Germany) at a final concentration of 20%. Samples were then stored at −80°C and transported on dry ice to the Friedrich-Loeffler-Institut, Germany. One hundred microlitres of an overnight culture was then plated on CHROMagar Orientation (MastDiagnostica GmbH, Reinfeld, Germany) supplemented with 2 µg/mL cefotaxime, and incubated overnight at 37°C. Cefotaxime-resistant colonies of *E. coli* and other *Enterobacterales* were subcultured until pure cultures were obtained and selected for further characterization. All isolates were stored at −80°C in LB broth and glycerol at a final concentration of 20%.

### WGS

Total DNA was extracted using the MasterPure DNA Purification Kit for Blood, v. 2 (Lucigen, Middleton, WI, USA), according to the manufacturer’s instructions. DNA was then quantified fluorometrically using the Qubit 4 Fluorometer and the corresponding dsDNA HS Assay Kit (Thermo Fisher Scientific, Waltham, MA, USA). DNA was shipped to SeqCenter in Pittsburgh, PA, USA, and sequenced on an Illumina NextSeq 2000 after library preparation using the Illumina DNA Prep kit and IDT 10 bp UDI indices (Illumina, San Diego, CA, USA), resulting in 2 × 151 bp reads. Demultiplexing, quality control and adapter trimming were performed using bcl-convert v. 3.9.3 (https://support-docs.illumina.com/SW/BCL_Convert/Content/SW/FrontPages/BCL_Convert.htm).

### Sequence assembly and genomic analyses

Raw sequences were processed using BBDuk from BBTools v. 38.95 (https://sourceforge.net/projects/bbmap/) to perform adapter trimming, filtering for possible PhiX contamination, and additional quality and polymer trimming. Quality control of the delivered (raw) and processed (trimmed) reads was performed using FastQC v. 0.11.9 (https://www.bioinformatics.babraham.ac.uk/projects/fastqc/). Trimmed reads were then assembled into contiguous sequences (contigs) using Shovill v. 1.1.0 (https://github.com/tseemann/shovill) with SPAdes v. 3.15.3.^6^ An additional polishing step was performed by first mapping the trimmed reads to the assembly using BWA v. 0.7.17.^7^ The alignment files were then converted to binary format, sorted, and duplicate reads were marked using SAMtools v. 1.14.^8^ Finally, the draft contigs were corrected with Pilon v. 1.24.^9^ Genome completeness and contamination were assessed using CheckM v. 1.1.3.^10^ For annotating the draft assembly, we used Prokka v. 1.14.6.^11^ Genomic analyses such as MLST and antibiotic and heavy metal resistance gene detection were performed using mlst v. 2.19.0 (https://github.com/tseemann/mlst; with the PubMLST^12^ database and Enterobase,^13^ and AMRFinderPlus v. 3.10.30 with database v. 2022-05-26.1.^14^

### Phenotypic antimicrobial susceptibility testing

MICs were determined with the automated VITEK 2 system (AST-N428 and AST-XN24; bioMérieux, Marcy l’Étoile, France), according to the manufacturer’s instructions. Susceptibility to cefiderocol was assessed by disc diffusion tests using cefiderocol 30 μg discs (Mast Diagnostics, Merseyside, UK). Isolates assigned to technical uncertainty (*Enterobacterales*, 18–22 mm^15^) were re-analysed using a commercial broth microdilution kit (ComASP, Liofilchem, Waltham, MA, USA) according to the manufacturer’s instructions. All results were interpreted according to the published breakpoints and guidelines of EUCAST.^15^

## Results and discussion

From 121 isolates overall, the most prevalent species was *E. coli*, accounting for 70.25% (85/121), followed by *K. pneumoniae sensu stricto* (referred to as *K. pneumoniae*; 23.14%; 28/121), *Enterobacter cloacae* (4.13%; 5/121), and one off-targeted *Pseudomonas aeruginosa* isolate (0.83%; 1/121). AMR gene analysis revealed that all isolates carried ESBL genes with *bla*_CTX-M-15_ as the dominant type (94.21%; 114/121). Alarmingly, 23 (19.01%; 23/121) isolates carried genes associated with carbapenem resistance in addition. Specifically, 15 *E. coli*, six *K. pneumoniae* and two *E. cloacae* were positive for carbapenemase-encoding genes, with *bla*_OXA-181_ being the most prevalent (69.57%; 16/23), followed by *bla*_NDM-5_ (21.74%; 5/23) and *bla*_OXA-48_ (8.70%; 2/23).

We performed antimicrobial susceptibility testing for these isolates to verify carbapenem resistance phenotypes. In addition, we evaluated susceptibility to the recently approved and important last-resort siderophore cephalosporin antibiotic cefiderocol (Table 1).

**Table 1.** Overview of the CPE and their phenotypic and genotypic properties

Isolate	Species	ST	Carbapenemase gene^a^	Phenotype								
				ETP		IPM		MEM		FDC		
				MIC (mg/L)	S/I/R^b^	MIC (mg/L)	S/I/R^b^	MIC (mg/L)	S/I/R^b^	ZD [mm]	MIC (mg/L)	S/R^b^
PBIO3888	Eco	1588	*bla*_NDM-5_	≥8	R	≥16	R	≥16	R	19^c^	8^c^	R
PBIO3896	Eco	410	*bla*_NDM-5_	≥8	R	≥16	R	≥16	R	20^c^	2^c^	S
PBIO3897	Eco	44	*bla*_OXA-181_	1	R	1	S	0.5	S	24	ND	S
PBIO3903	Eco	1588	*bla*_NDM-5_	≥8	R	≥16	R	≥16	R	19^c^	4^c^	R
PBIO3919	Kpn	2668	*bla*_OXA-181_	2	R	4	I	2	S	28	ND	S
PBIO3920	Kpn	25	*bla*_OXA-181_	2	R	4	I	2	S	28	ND	S
PBIO3921	Ecl	171	*bla*_OXA-48_	2	R	4	I	4	I	24	ND	S
PBIO3922	Kpn	2668	*bla*_OXA-181_	≥8	R	4	I	2	S	29	ND	S
PBIO3923	Kpn	25	*bla*_OXA-181_	2	R	4	I	2	S	27	ND	S
PBIO3924	Ecl	171	*bla*_OXA-48_	1	R	4	I	4	I	24	ND	S
PBIO3925	Kpn	25	*bla*_OXA-181_	1	R	2	S	2	S	28	ND	S
PBIO3927	Kpn	25	*bla*_OXA-181_	2	R	2	S	2	S	28	ND	S
PBIO3928	Eco	410	*bla*_OXA-181_	4	R	0.5	S	0.5	S	22	ND	S
PBIO3929	Eco	410	*bla*_OXA-181_	2	R	0.5	S	2	S	23	ND	S
PBIO3930	Eco	6359	*bla*_OXA-181_	1	R	1	S	≤0.25	S	25	ND	S
PBIO3931	Eco	410	*bla*_OXA-181_	2	R	0.5	S	2	S	22	ND	S
PBIO3933	Eco	410	*bla*_OXA-181_	4	R	1	S	2	S	23	ND	S
PBIO3934	Eco	1588	*bla*_NDM-5_	≥8	R	≥16	R	≥16	R	20^c^	2^c^	S
PBIO3935	Eco	410	*bla*_OXA-181_	4	R	0.5	S	2	S	23	ND	S
PBIO3936	Eco	410	*bla*_NDM-5_	≥8	R	8	R	≥16	R	26	ND	S
PBIO3937	Eco	6359	*bla*_OXA-181_	4	R	0.5	S	2	S	23	ND	S
PBIO3938	Eco	6359	*bla*_OXA-181_	2	R	1	S	2	S	29	ND	S
PBIO3939	Eco	410	*bla*_OXA-181_	≥8	R	1	S	2	S	22	ND	S

Ecl, *E. cloacae*; Eco, *E. coli*, ETP, ertapenem; FDC, cefiderocol; I, susceptible, increased exposure; IPM, imipenem; Kpn, *K. pneumoniae*; MEM, meropenem; ND, not determined; R, resistant; S, susceptible, standard dosing regimen; ZD, zone diameter.

^a^Predictions for carbapenemase genes are based on alignments of sequences from the AMRFinderPlus database.^14^ Default settings of identity (use a curated threshold if it exists, and ≥90.0% otherwise) and coverage (≥50.0%).

^b^Interpretation categories according to EUCAST guidelines.^15^

^c^For isolates with zone diameters within the range of technical uncertainty (*Enterobacterales*, 18–22 mm^15^), the MIC of cefiderocol was determined by broth microdilution.

The *bla*_OXA-181_-positive isolates were only resistant to ertapenem, highlighting the weak hydrolytic activity of this OXA-48-like carbapenemase against carbapenems.^16^ In contrast, isolates carrying *bla*_NDM-5_ or *bla*_OXA-48_ showed higher MICs of imipenem and/or meropenem. Notably, the CPE were predominantly associated with internationally recognized high-risk clonal lineages, such as *E. cloacae* ST171, *E. coli* ST410 and ST1588, and *K. pneumoniae* ST25. Many studies have consistently demonstrated that successful clonal lineages carry multiple AMR determinants, can be rapidly transmitted among and persist in different host species and ecosystems, may cause severe disease in animals and humans, and are globally distributed (e.g. the study by Eger *et al.*^17^). Environmental implications of AMR emergence predominantly originate from high-resource settings, leaving a significant knowledge gap in LMICs. The facilitation of AMR occurs through the discharge of sewage and antimicrobial residues into the environment and the inadequate treatment of human and animal waste. In Ghana, like in other countries in SSA, the drainage systems comprise open drains and street gutters fed from various sources including households, hospitals and industries, subsequently contaminating the environment. In fact, we frequently observed private household sewage and excrement from livestock and feral domestic animals near the sampling spots. Reducing environmental contamination with AMR bacteria primarily includes public education programmes to not only raise awareness about the risks associated with untreated effluent discharge into the environment but also regarding the usage of lake and river water for domestic purposes. In addition, improving basic sanitation and increasing the number and efficacy of sewage treatment plants is crucial.

To the best of our knowledge, this is the first study identifying cefiderocol-resistant *Enterobacterales* in surface water from SSA. Both resistant isolates, PBIO3888 and PBIO3903, were collected shortly after the international approval and clinical use of cefiderocol in 2019. Interestingly, the corresponding sampling location is at the Kumasi Zoo compound, which, although access-restricted, receives wastewater from the zoo enclosures, visitors and the nearby central marketplace. Recent studies have suggested several mechanisms contributing to cefiderocol resistance, including gene alterations in the iron transport pathway and nutrient uptake (e.g. *cirA* and *ompC*).^18^ However, a BLAST analysis of the amino acid sequences of CirA (UniProt accession P17315), OmpF (UniProt accession P02931) and OmpC (UniProt accession P06996), using *E. coli* K-12 as a reference, did not reveal any potential resistance-mediating mutations in our isolates. This suggests that cefiderocol resistance may be attributed to overexpression of the NDM-5 carbapenemase.

Ghana faces the challenge of environmental contamination with heavy metals, particularly those associated with illegal gold mining activities.^19^ Heavy metals in the environment may co-select for AMR in bacteria, which is caused by either single mechanisms conferring cross-resistance to both antibiotics and heavy metals, and/or the occurrence of different resistance determinants located on the same genetic element.^20^ Except for seven of the *E. coli* isolates in this study, all CPE were positive for genes associated with multi-metal RND efflux pump activity (*silABCEFPRS*), which typically confer resistance to various heavy metals, including silver. Genes involved in copper resistance (*pcoABCDRS*; 17/24), followed by arsenic resistance (*arsD*; 8/24), mercury resistance (*merRT*; 7/24) and tellurium resistance (*terD*; 4/24) also occurred. Consequently, additional assessment of not only heavy metal resistance determinants but also heavy metal residues should be an elementary component of surface water surveillance approaches.^5^

Even though this is a proof-of-concept study, it could have been improved by considering more sampling sites, a longer sampling period, and by obtaining relevant metadata including weather conditions and physicochemical water parameters during sampling. These issues will be addressed in a follow-up, large-scale study.

## Conclusions

In conclusion, we not only found ESBL-PE and CPE but also, more importantly, two isolates resistant to the recently approved antibiotic cefiderocol in surface water samples in Ghana, also indicating the potential for transmission to humans and animals. Our study suggests that further exploration of transmission pathways related to environmental contamination is warranted.

## Data Availability

The data for this study have been deposited in the European Nucleotide Archive (ENA) at EMBL-EBI under accession number PRJEB64632 (https://www.ebi.ac.uk/ena/browser/view/PRJEB64632).
